# Medical Maximizing Preferences and Beliefs About Cancer Among US Adults

**DOI:** 10.1001/jamanetworkopen.2024.17098

**Published:** 2024-06-14

**Authors:** Alexander S. Chiu, Ines Hoxha, Catherine B. Jensen, Megan C. Saucke, Susan C. Pitt

**Affiliations:** 1 Department of Surgery, University of Wisconsin School of Medicine and Public Health, Madison; 2 Department of Surgery, University of Michigan, Ann Arbor

## Abstract

**Importance:**

Medical overutilization contributes to significant health care expenditures and exposes patients to questionably beneficial surgery and unnecessary risk.

**Objectives:**

To understand public attitudes toward medical utilization and the association of these attitudes with beliefs about cancer.

**Design, Setting, and Participants:**

In this cross-sectional survey study conducted from August 26 to October 28, 2020, US-based, English-speaking adults were recruited from the general public using Prolific Academic, a research participant platform. Quota-filling was used to obtain a sample demographically representative of the US population. Adults with a personal history of cancer other than nonmelanoma skin cancer were excluded. Statistical analysis was completed in July 2022.

**Main Outcome and Measures:**

Medical utilization preferences were characterized with the validated, single-item Maximizer-Minimizer Elicitation Question. Participants preferring to take action in medically ambiguous situations (hereafter referred to as “maximizers”) were compared with those who leaned toward waiting and seeing (hereafter referred to as “nonmaximizers”). Beliefs and emotions about cancer incidence, survivability, and preventability were assessed using validated measures. Logistic regression modeled factors associated with preferring to maximize medical utilization.

**Results:**

Of 1131 participants (mean [SD] age, 45 [16] years; 568 women [50.2%]), 287 (25.4%) were classified as maximizers, and 844 (74.6%) were classified as nonmaximizers. Logistic regression revealed that self-reporting very good or excellent health status (compared with good, fair, or poor; odds ratio [OR], 2.01 [95% CI, 1.52-2.65]), Black race (compared with White race; OR, 1.88 [95% CI, 1.22-2.89]), high levels of cancer worry (compared with low levels; OR, 1.62 [95% CI, 1.09-2.42]), and overestimating cancer incidence (compared with accurate estimation or underestimating; OR, 1.58 [95% CI, 1.09-2.28]) were significantly associated with maximizing preferences. Those who believed that they personally had a higher-than-average risk of developing cancer were more likely to be maximizers (23.6% [59 of 250] vs 17.4% [131 of 751]; *P* = .03); this factor was not significant in regression analyses.

**Conclusions and Relevance:**

In this survey study of US adults, those with medical maximizing tendencies more often overestimated the incidence of cancer and had higher levels of cancer-related worry. Targeted and personalized education about cancer and its risk factors may help reduce overutilization of oncologic care.

## Key Points

**Question:**

What are the attitudes of the US public toward health care utilization, and what is the association of these attitudes with beliefs about cancer?

**Findings:**

In this survey study of 1131 adults, individuals with a preference for taking action in medically ambiguous situations (“medical maximizing”) more often overestimated the incidence of cancer and had higher levels of cancer-related worry.

**Meaning:**

This study suggests that educational efforts to reduce patient-associated medical overutilization across the cancer continuum should account for emotional concerns about cancer and beliefs about the risk of developing cancer.

## Teaser

This survey study analyzes a representative sample of US adults regarding their preferences for maximizing utilization of medical tests and interventions and the association of these attitudes with beliefs about cancer.

## Introduction

Medical and surgical overtreatment is a significant problem in the US that exposes patients to unnecessary risks and results in more than $200 billion of health care waste each year.^1^ Countless examples in the literature document frequent use of unneeded diagnostic tests, medical treatments, and invasive interventions that provide no proven clinical benefit.^2,3,4^ Although the cause of overtreatment is multifactorial and includes physician- and system-level factors—such as fear of malpractice, lack of care coordination, duplicate tests, and diminished time with patients—patient preference has also been cited as a major contributor to overtreatment, yet is less often studied.^5,6,7^ In a survey of more than 2000 physicians, the second most commonly cited reason for overtreatment, after fear of malpractice, was pressure from patients to order tests and treatments.^8^ Part of this push from patients comes from a common perception that more care means better care, combined with increased satisfaction when patients perceive something proactive is “being done” for their health, regardless of utility.^9,10^

Management of cancer is particularly susceptible to medical overuse and unnecessary care and occurs at multiple time points, including screening, diagnosis, treatment, and surveillance.^7,11,12^ The emphasis on shared decision-making, individualized care at each phase along the cancer care continuum, and the expansion of potential treatment modalities with their own unique benefits and risks has increased the importance of incorporating patient preference into oncologic medical decision-making. Further challenging the decision-making process is that the diagnosis or potential diagnosis of cancer has a major emotional effect on patients that can influence their preference and color their decision-making.^13^

The Maximizer-Minimizer Elicitation Question (MM1) is a validated, single-question evaluation that was developed to measure medical utilization preferences of the general public.^14^ This tool helps identify patients who desire an aggressive or “maximizing” approach to medical care (hereafter referred to as “maximizers”). Studies have shown that maximizers are more likely to want unnecessary or low-value tests and interventions, potentially leading to overutilization of health care.^15,16^ Understanding patients’ maximizing preferences is a critical first step in addressing patient desires for overtreatment in cancer. This study sought to better understand the general public’s attitudes and beliefs about cancer and how these beliefs are associated with their medical utilization tendencies. We hypothesized that individuals with less knowledge and greater worry or fear of cancer would be more likely to have medical maximizing tendencies that lead to overtreatment. A better understanding of patients’ cancer-related beliefs provides the background for potential interventions to reduce patient preferences for unnecessary care.

## Methods

### Study Population

In this survey study, participants were recruited from the US general public using Prolific Academic, a research participant platform that maintains a panel of more than 130 000 people from around the world who voluntarily participate in behavioral research.^17,18^ This platform has been shown to provide valid, reliable, and reproducible results with higher data quality than other crowdsourcing platforms.^17,18^ Inclusion criteria for this study were English-speaking adults aged 18 years or older, located in the US, and without a personal history of cancer other than nonmelanoma skin cancer, such as basal or squamous cell cancer (because these low-risk cutaneous malignant neoplasms rarely result in death). The exclusion of participants with a cancer history allowed examination of a more homogenous, cancer-naive population. Participants were recruited between August 26 and October 28, 2020. We used quota filling to recruit a sample that was demographically representative of the US population at large when considering age, gender identity, and participant-reported race and ethnicity (based on 2010 US Census Bureau population group estimates).^19,20,21^ Race and ethnicity data were assessed in this study to examine potential differences in medical maximizing preferences and beliefs about cancer across racial and ethnic groups. Our response goal was 1200 based on published literature^22^; population estimates were not available for our primary outcome measure to inform a power calculation. Respondents were incentivized to participate through the payment of a nominal monetary reward of $1.46, which equated to $15 per hour based on the mean time to complete the survey instrument. Written informed consent was obtained from all participants. This study was approved by the institutional review boards at the University of Wisconsin and University of Michigan. This study followed the American Association for Public Opinion Research (AAPOR) reporting guideline.

### Survey Design

The survey instrument was developed and reviewed with subject matter experts, cognitively tested, and piloted for participant ease and understanding prior to distribution. The complete survey instrument was 43 questions and assessed worry and fear about cancer, general beliefs and estimations about cancer, basic demographics, and medical maximizing tendencies (eMethods 1 and 2 in Supplement 1). Qualtrics software was used for survey administration.

To measure medical maximizing tendencies, participants were asked the validated, single-item MM1. This question asks the respondent their preferred course of action in situations in which the need for medical action is not clear.^14^ Response options are on a 6-point Likert scale ranging from strongly lean towards watching and waiting to strongly lean towards taking action. For analysis, those who answered strongly lean or lean toward taking action were considered maximizers, and were compared with those who somewhat leaned toward taking action, somewhat leaned toward waiting and seeing, and leaned or strongly leaned toward waiting and watching (collectively referred to as nonmaximizers).^23^ Prior research has shown maximizers to be individuals who typically seek help from physicians and receive medical treatment regardless of its proven efficacy, whereas minimizers generally prefer not receiving interventions unless absolutely necessary.^15,23,24^ The dichotomization of maximizer and nonmaximizer was found to be most clinically useful, and the classification of maximizers was in line with prior research.^23^

In addition, participants were asked questions from the National Cancer Institute Health Information National Trends Survey (HINTS), which collects information on the US public’s use and understanding of cancer-related information.^25^ The questions asked respondents were about their degree of agreement about the curability (“Cancer is an illness that when detected early can typically be cured”) and preventability (“There’s not much you can do to lower your chances of getting cancer”) of cancer. Salience of cancer mortality was assessed by asking if participants “automatically thought of death” when they thought of cancer. These 3 questions were assessed using a 4-point Likert scale ranging from strongly agree to strongly disagree and were dichotomized into affirmative (strongly agree and agree) and negative (strongly disagree and disagree) answers for analysis.^26,27^

The survey also included questions about respondents’ levels of worry (the emotional reaction to the threat of cancer) and fear (the physical manifestation of a negative emotion often seen with cancer diagnosis) about cancer using single-item, validated questions from the HINTS. Fear was assessed with the statement “The thought of cancer scares me” and was answered with a 5-point Likert scale from strongly agree to strongly disagree. Worry was assessed with the question “How often do you worry about getting cancer?” and was also answered with a 5-point Likert scale (never, rarely, sometimes, often, and all of the time). Answers for both were dichotomized for analysis as high fear (strongly agree and agree) or low fear (neither agree or disagree, disagree, and strongly disagree) and frequent worry (often and all of the time) or infrequent worry (never, rarely, and sometimes).

Participants’ belief of their personal risk of cancer in general was assessed using a 5-point Likert scale ranging from very low to very high. In addition, participants were asked their estimates of the general incidence and 5-year survivability of cancer in the US. For these questions, a range of percentages were presented (0%-9%, 10%-29%, 30%-49%, 50%-69%, 70%-89%, and 90%-100%), and answers were considered accurate, overestimates, or underestimates in comparison with recent established epidemiologic rates (correct answers being 38%-40% for incidence and 78%-80% for 5-year survivability).^28^

Finally, 2 attention-check questions were included at different places in the survey. The first attention-check question instructed respondents to choose a specific answer to gauge if they were paying attention, and respondents were excluded if they failed to do so. The second attention-check question asked about their risk of breast and prostate cancer; women who indicated they had a risk of prostate cancer and men who indicated they had a high or very high risk of breast cancer were excluded. Attention checks are recommended in surveys of participant pools to ensure data quality.

### Statistical Analysis

Frequencies of responses were calculated and are presented as simple percentages. A priori levels of significance were set at *P* < .05, and hypothesis tests were 2 sided. Survey response comparisons between maximizer and nonmaximizer groups were calculated using the χ^2^ test for categorical variables and the *t* test for continuous variables. Multivariable logistic regression was conducted to determine the demographics and beliefs that were most strongly associated with medical maximizing tendencies. The model was conducted using a backward selection model, with α < .05 considered as significant. All demographic variables (age, gender, race and ethnicity, and educational level) and survey questions thought to potentially be associated with maximizing preferences, based on author consensus, were included. Statistical analysis was conducted using SAS, version 9.4 (SAS Institute Inc).

## Results

A total of 1205 individuals completed the survey, of whom 1131 met inclusion criteria and passed both attention checks (93.9% eligibility). Respondents had a mean (SD) age of 45 (16) years, 568 (50.2%) were women, 549 (48.5%) were men, 70 (6.2%) were Asian or Other Pacific Islander, 140 (12.4%) were Black or African American, 50 (4.4%) were Hispanic, and 836 (73.9%) were White (Table). Among eligible respondents, 287 (25.4%) were classified as maximizers. There were no significant differences between medical maximizers and nonmaximizers in terms of age distribution, sex, race and ethnicity, or marital status (Table). Educational levels between groups were also similar, although maximizers were significantly less likely than nonmaximizers to have graduate or professional degrees (21 of 287 [7.3%] vs 117 of 844 [13.9%]; *P* = .04). In addition, maximizers were more likely than nonmaximizers to rate their health status as excellent or very good (152 of 287 [53.0%] vs 323 of 844 [38.3%]; *P* < .001). Both groups had similarly high rates of having a close friend or family member with a history of cancer (217 of 287 [75.6%] maximizers vs 647 of 844 [76.7%] nonmaximizers; *P* = .26).

**Table.** Demographics of the Study Population by Medical Maximizing Preferences

Characteristic	No. (%)			*P* value
	Maximizers (n = 287)	Nonmaximizers (n = 844)	Total (N = 1131)	
Sex				
Female	148 (51.6)	420 (49.8)	568 (50.2)	.82
Male	135 (47.0)	414 (49.8)	549 (48.5)	
Nonbinary or transgender	4 (1.4)	10 (0.4)	14 (1.3)	
Age, y				
<40	121 (42.2)	343 (40.6)	464 (41.0)	.69
40-64	127 (44.3)	397 (47.0)	524 (46.3)	
≥65	39 (13.6)	104 (12.3)	143 (12.6)	
Race and ethnicity				
Asian or Other Pacific Islander	14 (4.9)	56 (6.6)	70 (6.2)	.10
Black or African American	49 (17.1)	91 (10.8)	140 (12.4)	
Hispanic	11 (3.8)	39 (4.6)	50 (4.4)	
White	204 (71.1)	632 (74.9)	836 (73.9)	
Other or multiracial^a^	9 (3.1)	25 (3.0)	34 (3.0)	
Educational level				
Some high school, less or high school graduate, or GED certification	102 (35.5)	275 (32.6)	377 (33.3)	.04
Some college or associate’s degree	104 (36.2)	298 (35.3)	402 (35.5)	
Bachelor’s degree	60 (20.9)	151 (17.9)	211 (18.7)	
Graduate or professional degree	21 (7.3)	117 (13.9)	138 (12.2)	
Marital status				
Married or living as married	146 (50.9)	423 (50.1)	569 (50.3)	.98
Divorced, widowed, or separated	41 (14.3)	122 (14.5)	163 (14.4)	
Single, never been married	100 (34.8)	299 (35.4)	399 (35.3)	
Health status				
Excellent or very good	152 (53.0)	323 (38.3)	475 (42.0)	<.001
Good	79 (27.5)	349 (41.4)	428 (37.8)	
Fair or poor	56 (19.5)	172 (20.4)	228 (20.2)	
History of close family member or friend with cancer	217 (75.6)	647 (76.7)	864 (76.4)	.26
Personal belief of likelihood of developing cancer in the future				
Very low, somewhat low, or moderate	230 (80.1)	702 (83.2)	932 (82.4)	.33
Somewhat high or very high	57 (19.9)	140 (16.6)	197 (17.4)	

Abbreviation: GED, General Education Development.

^a^ “Other” includes Caribbean, Jewish American, North African Middle Eastern, and West Indian.

### Cancer Statistics and Maximizing Tendencies

**Figure 1.**
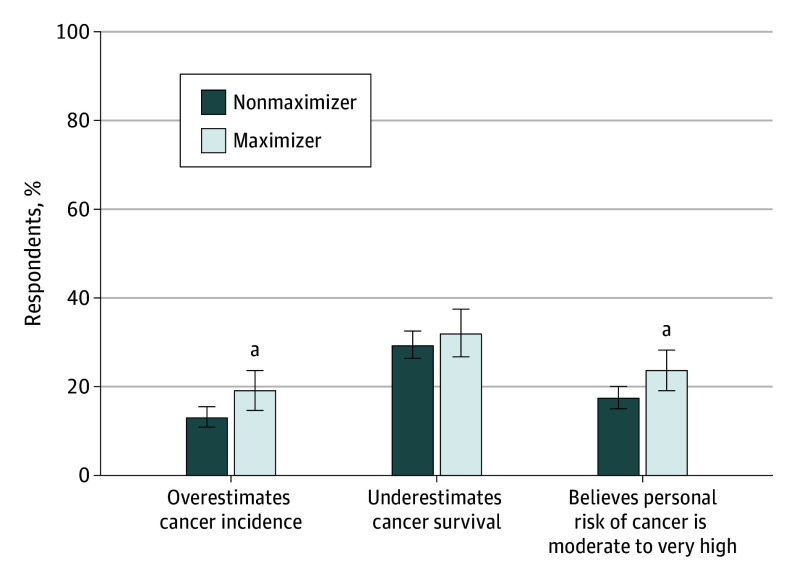
Beliefs About Cancer Incidence, Survival, and Personal Risk by Medical Maximizing Preferences ^a^*P* < .05.

When asked about their beliefs on the incidence of cancer in general, medical maximizers were more likely to provide an overestimate compared with nonmaximizers (55 of 287 [19.2%] vs 110 of 844 [13.0%]; *P* = .01) (Figure 1). However, when asked about the survivability of cancer, maximizers and nonmaximizers underestimated the overall 5-year cancer survival at the same rate (248 of 844 [29.4%] vs 92 of 287 [32.1%]; *P* = .39), and no respondents overestimated the survivability of cancer. Maximizers more frequently reported that they personally had a higher risk of cancer than average compared with nonmaximizers (59 of 250 [23.6%] vs 131 of 751 [17.4%]; *P* = .03) (Figure 1).

### Cancer Beliefs and Maximizing Tendencies

**Figure 2.**
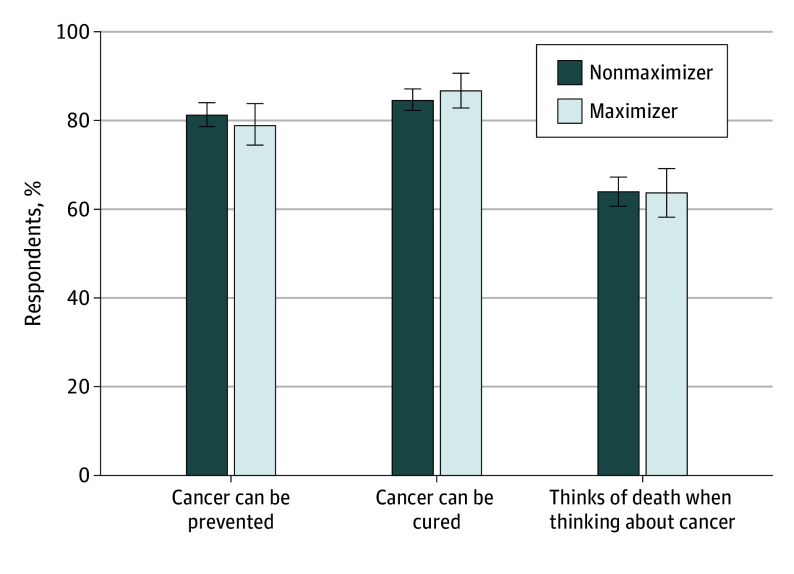
Beliefs About Cancer Preventability, Curability, and the Salience of Mortality by Medical Maximizing Preferences

**Figure 3.**
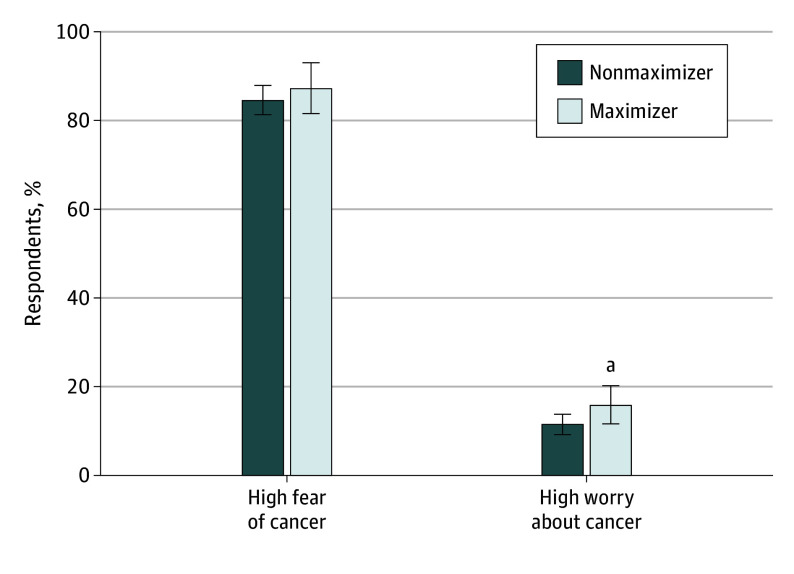
Fear and Worry About Cancer by Medical Maximizing Preferences ^a^*P* < .05.

Respondents were also asked if they believed that cancer could be prevented and cured, and if they automatically thought of death when thinking of cancer, which is a measure of mortality salience with a cancer diagnosis. For each of these questions, there was no significant difference in the proportion of respondents who agreed with those statements (Figure 2). Similarly, when asked about their fear of cancer, there was no significant difference betweein the percentage of maximizers and the percentage of nonmaximizers reporting high fear (714 of 843 [87.4%] vs 251 of 857 [84.7%]; *P* = .25) (Figure 3). However, when asked about cancer-related worry, maximizers were more likely than nonmaximizers to report high levels of worry (45 of 287 [15.7%] vs 95 of 844 [11.3%]; *P* = .05).

### Factors Associated With Maximizing Tendencies

**Figure 4.**
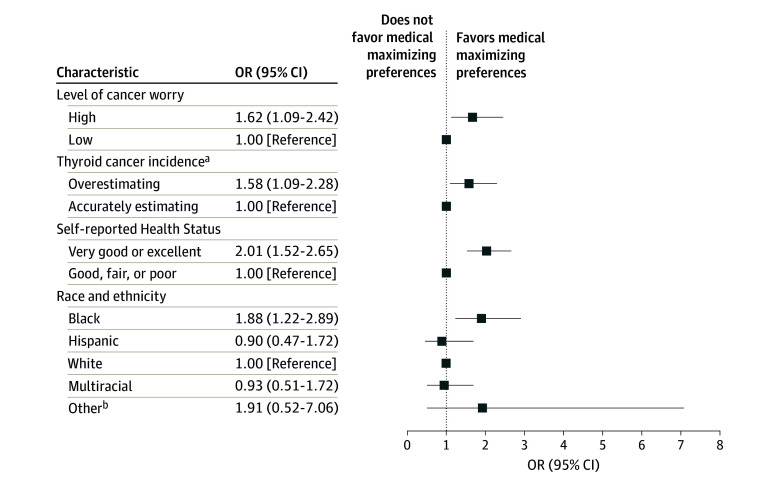
Logistic Regression for Characteristics Associated With Medical Maximizing Preferences OR indicates odds ratio. ^a^No respondents underestimated thyroid cancer. ^b^Includes Caribbean, Jewish American, North African Middle Eastern, and West Indian.

Logistic regression of demographics and respondent beliefs about cancer demonstrated that race and ethnicity, self-reported health status, cancer-related worry, and beliefs about cancer incidence were significantly associated with medical maximizing tendencies. The strongest associations were self-reporting a very good or excellent health status (compared with good, fair, or poor; odds ratio [OR], 2.01 [95% CI, 1.52-2.65]) and Black race (compared with White race; OR, 1.88 [95% CI, 1.22-2.89]) (Figure 4). Overestimating the incidence of cancer (compared with accurate estimation or underestimating; OR, 1.58 [95% CI, 1.09-2.28]) and high cancer worry (compared with low cancer worry; OR, 1.62 [95% CI, 1.09-2.42]) were also associated with being a maximizer.

## Discussion

This study explored the attitudes and beliefs of the US public regarding cancer and how these attitudes and beliefs are associated with preferences for medical utilization. One important finding is that one-fourth of respondents in this nationally distributed survey were identified as medical maximizers. The prevalence of medical maximizing preferences has previously been undefined at a scale such as this study, to our knowledge.

Knowing the magnitude of potential patients with high medical maximizing preferences is significant, as maximizers have been shown to be more likely to pursue health care with questionable benefit, likely contributing to the significant amount of overtreatment seen in the US health system.^15,23^ In a study by Scherer et al,^15^ male participants were asked about their interest in obtaining prostate-specific antigen testing before and after being provided information about its risks and benefits. Those with maximizing tendencies were significantly more likely to continue to want to pursue diagnostic testing compared with the rest of the cohort. Similarly, in a study by Mott et al^23^ of women older than 70 years for whom the aggressive treatment of breast cancer has questionable benefits, those with medical maximizing preferences were more likely to want to pursue more invasive therapies, even after being informed these therapies carry potentially excessive unnecessary risks. In the study by Mott et al,^23^ 30% of those surveyed were classified as maximizers based on their answers to the MM1; however, that study focused on a specific population—elderly women. In comparison, the data presented in the present study provide a broader context for the potential association of patient preference for medical maximizing with the overall overutilization of medical services in the US.

The results of the present study also showed that overestimating the incidence of cancer and having high cancer-related worry were strongly associated with preferring to maximize medical care. Previous studies have shown that overestimation of cancer incidence is common. For example, 1 study demonstrated that nearly half the US public erroneously believes that cancer is more prevalent than heart disease.^29^ Believing cancer is common or personal increased risk of developing cancer likely leads to increased worry about cancer, which were both significantly associated with maximizing tendencies in this study. This finding is in line with previous research that has shown that high levels of cancer worry are associated with increased utilization of screening tests, which can be positive in many scenarios, such as greater adherence to guideline-concordant screening recommendations, but can lead to overutilization in others.^30^ Although we do not know whether overestimation of cancer incidence leads to maximizing preferences (it is an area that needs further investigation), these associations suggest that directed education for the public focusing on the true incidence of cancer and tailored messaging toward those who are medical maximizers may be effective approaches to overcome patient-associated medical overutilization along the continuum of cancer screening, diagnosis, treatment, and surveillance. Prior work suggests that tailored information and patient education tools are viable strategies to address overutilization of cancer-related health care.^31,32^

Although medical maximizers were more likely to overestimate cancer incidence than nonmaximizers, the 2 groups had similar rates of accurately estimating the survivability of cancer in general. One may hypothesize that the concern of death underlies the tendency to maximize medical care, and that maximizers might be more likely than nonmaximizers to believe cancer is deadly. However, both groups had similar beliefs about 5-year cancer survival, suggesting that the threat of cancer coupled with the emotional effect and stigma of a cancer diagnosis may be more associated with behavior than the threat of death. Another hypothesis is that higher fear of cancer may contribute to the overestimation of cancer incidence by medical maximizers; however, in both groups, more than 80% of respondents had high fear of cancer. Prior studies support this idea and have shown that, while patients are quite preoccupied with cancer, they are often less focused on mortality. For example, in the original US HINTS survey, 45% of participants reported spending time seeking out information on cancer, yet only 2% of them looked up information on prognosis and 9% on cure rates.^33^ In all, these findings suggest that focused messaging on cancer incidence and patients’ personal risk of developing cancer may be an effective strategy in trying to reduce cancer-related overutilization.

Another important finding of this study is that Black respondents were more likely than White respondents to have medical maximizing tendencies. This result is consistent with another study evaluating medical maximizing preferences and thyroid cancer surveillance, in which 18% of thyroid cancer survivors in the maximizer group were Black compared with 6% of the nonmaximizer group.^24^ This finding also echoes what is seen with end of life care, as Black patients have been found to be more likely to pursue aggressive treatments and less likely to utilize hospice care.^34^ Qualitative studies have found that cultural and religious factors play a role in these preferences.^35^ Factors such as mistrust of the medical system stemming from a history of structural racism and poor clinician communication have further been shown to contribute to preferences for more aggressive treatment at the end of life.^36^ The historical context and continued mistreatment of those who identify as Black in the US health care system cannot be understated. Resulting disparities lead to mistrust and the increased preference for medical maximizing among Black participants found in this study.

This racial difference in medical maximizing preference may not translate into increased unnecessary treatment or overutilization. A systematic review of the racial and ethnic differences in overtreatment showed that White patients more often receive overtreatment.^37^ These findings highlight existing health disparities and a discordance in the preferences of patients of racial and ethnic minority groups with respect to their preference of taking action in medically ambiguous situations and their actual lack of receipt of this treatment. These findings may also suggest that maximizing tendencies in some groups promote equitable care as a result of patients advocating for themselves. These findings highlight the multifactorial causes of overtreatment. Going forward, it is essential that patient-directed interventions and education to reduce overtreatment are culturally sensitive.^38,39^

### Limitations

Our study had a number of limitations that should be considered. First, because Prolific is an opt-in service, those who join to answer surveys may not be fully representative of the population at large. They also by definition have internet access, which introduces selection bias. Second, the survey did not ask about participants’ comorbidities beyond their cancer history and perceived health status. Participants’ medical history may influence their medical utilization tendencies. In addition, participants were limited to English-speaking adults without a history of cancer, potentially missing cultural differences among non-English speaking populations or the association of a prior cancer diagnosis with medical utilization tendencies. Although our study assessed educational level and general health status, other unmeasured socioeconomic variables may influence health care utilization unrelated to medical maximizing tendencies.^40^ Another potential limitation is the overlap and correlation between the constructs of fear and worry, which may have affected how participants responded to related questions and the extent of our conclusions.

## Conclusions

This survey study found that those who prefer medical maximizing are more likely to be Black; have high cancer-related worry; overestimate the incidence, but not survival rates, of cancer; and self-report a very good or excellent health status. These findings indicate that educational efforts to reduce patient-associated medical overutilization across the cancer continuum should account for racial differences, emotional concerns about cancer, and beliefs about the risk of developing cancer.

## Notes

Supplement 1. **eMethods 1.** Survey Instrument **eMethods 2.** Summary of Survey Measures

Supplement 2.
Data Sharing Statement
