# Supplementary material for: Medical Maximizing Preferences and Beliefs About Cancer Among US Adults
Source: JAMA Netw Open. 2024 Jun 14;7(6):e2417098. doi: 10.1001/jamanetworkopen.2024.17098 (PMC11179133; doi:10.1001/jamanetworkopen.2024.17098)
Supplement: Supplement 1. — eMethods 1. Survey Instrument eMethods 2. Summary of Survey Measures [file jamanetwopen-e2417098-s001.pdf]

Supplementary Online Content

Chiu AS, Hoxha I, Jensen CB, Saucke MC, Pitt SC. Medical maximizing preferences and beliefs about cancer among US adults. *JAMA Netw Open.* 2024;7(7):e2417098. doi:10.1001/jamanetworkopen.2024.17098

eMethods 1. Survey Instrument
eMethods 2. Summary of Survey Measures

This supplementary material has been provided by the authors to give readers additional information about their work.

© 2024 Chiu AS et al. *JAMA Network Open.*

eMethods 1. Survey Instrument

General Cancer Questions: The following questions are about your thoughts related to cancer in general. For each question or statement, please check one answer.

Q.

	Never	Rarely	Sometimes	Often	All of the time
**Q. How often do you worry about getting cancer?**	☐	☐	☐	☐	☐

For the following question, please indicate how much do you agree or disagree with each of the following statements about cancer?

	Strongly Disagree	Disagree	Neither Agree nor Disagree	Agree	Strongly Agree
**Q. The thought of cancer scares me.**	☐	☐	☐	☐	☐

	Strongly Agree	Somewhat Agree	Somewhat Disagree	Strongly Disagree
**Q. Cancer is an illness that when detected early can typically be cured.**	☐	☐	☐	☐
**Q. When I think about cancer, I automatically think of death.**	☐	☐	☐	☐
**Q. There's not much you can do to lower your chances of getting cancer.**	☐	☐	☐	☐

Q. What would you estimate is the average person's likelihood of being diagnosed with cancer in their lifetime in the United States?

- ☐ Less than 10%
- ☐ 10 to 29%
- ☐ 30 to 49%
- ☐ 50 to 69%
- ☐ 70 to 89%
- ☐ More than 90%

Q. Overall, how many people who develop cancer in the United States do you think survive at least 5 years?

- ☐ Less than 10%
- ☐ 10 to 29%
- ☐ 30 to 49%
- ☐ 50 to 69%
- ☐ 70 to 89%
- ☐ More than 90%

© 2024 Chiu AS et al. *JAMA Network Open.*

Q. How likely do you think it is that you will develop cancer in the future? Would you say your chance of getting ______ is very low, somewhat low, moderate, somewhat high, or very high?

	Very Low	Somewhat Low	Moderate	Somewhat High	Very High
**Q. Cancer in general**	☐	☐	☐	☐	☐
**Q. Prostate cancer**	☐	☐	☐	☐	☐
**Q. Breast cancer**	☐	☐	☐	☐	☐

Demographics

Lastly, we want to learn more about you.

Q. Age:
[free text entry box] years

Q. How do you think of yourself?
- ☐ Female
- ☐ Male
- ☐ Not listed: ________________________

Q. What is your race/ethnicity? Select all that apply:
- ☐ White
- ☐ Black/African American
- ☐ Hispanic or Latino
- ☐ American Indian/Alaska Native
- ☐ Asian
- ☐ Native Hawaiian/Other Pacific Islander
- ☐ Not listed: ________________________

Q. What is the highest level of education you completed?
- ☐ Some High School or less
- ☐ High School Graduate/GED
- ☐ Some College or Associate's Degree
- ☐ Bachelor's Degree
- ☐ Graduate/ Professional Degree

Q.What is your marital status?
- ☐ Married
- ☐ Living as married or living with a romantic partner
- ☐ Divorced
- ☐ Widowed
- ☐ Separated
- ☐ Single, never been married

Q.Do you have any children?
- ☐ Yes
- ☐ No
- ☐ Not sure

© 2024 Chiu AS et al. *JAMA Network Open.*

Q. In general, would you say your health is…?

- ☐ Excellent
- ☐ Very good
- ☐ Good
- ☐ Fair
- ☐ Poor

Q. Sometimes, medical action is clearly necessary, and sometimes it is clearly NOT necessary. Other times, reasonable people differ in their beliefs about whether medical action is needed.

In situations where it's not clear, do you tend to lean towards taking action or do you lean towards waiting and seeing if action is needed?

Importantly, there is no "right" way to be.

	I strongly lean toward waiting and seeing 1	I lean toward waiting and seeing 2	I somewhat lean toward waiting and seeing 3	I somewhat lean toward taking action 4	I lean toward taking action 5	I strongly lean toward taking action 6
**Please answer on the 1-6 scale:**	☐	☐	☐	☐	☐	☐

Q. Has a close family member or friend of yours had cancer?

- ☐ Yes
- ☐ No
- ☐ Not sure

Attention checks:

Q. How much time did you spend looking up information about cancer while taking this survey?

- ☐ None
- ☐ Less than 5 minutes
- ☐ More than 5 minutes

© 2024 Chiu AS et al. *JAMA Network Open.*

eMethods 2. Summary of Survey Measures

Measure	Question:	Response Scale		Validation
Medical Maximizer Elicitation Question (MM1)	"In situations where it's not clear, do you tend to lean towards taking action or do you lean towards waiting and seeing if action is needed?"	6-point Likert scale	"strongly lean towards watching and waiting" to "strongly lean towards taking action"	Yes
Health Information National Trends Survey (HINTS) Curability	"Cancer is an illness that when detected early can typically be cured."	4-point Likert scale	"strongly agree" to "strongly disagree"	Yes
HINTS Preventability	"There's not much you can do to lower your chances of getting cancer."	4-point Likert scale	"strongly agree" to "strongly disagree"	Yes
HINTS Salience of Cancer Mortality	"When I think about cancer, I automatically think of death."	4-point Likert scale	"strongly agree" to "strongly disagree"	Yes
HINTS Level of Cancer Fear	"The thought of cancer scares me."	5-point Likert scale	"strongly agree" to "strongly disagree"	Yes
HINTS Level of CancerWorry	"How often do you worry about getting cancer?"	5-point Likert scale	"never," "rarely," "sometimes," "often," and "all of the time"	Yes
Belief of Personal Risk of Cancer	"How likely do you think it is that you will develop cancer in the future? Would you say your chance of getting ______ is very low, somewhat low, moderate, somewhat high, or very high?"	5-point Likert scale	"very low" to "very high"	No*
General Incidence of Cancer	"What would you estimate is the average person's likelihood of being diagnosed with cancer in their lifetime in the United States?"	Percentages ranges (0-9%, 10-29%, 30-49%, 50-69%, 70-89%, and 90-100%);	Answers were considered "accurate," "overestimates," or "underestimates" in comparison to 38-40%	No*
5-Year Survivability of Cancer	"Overall, how many people who develop cancer in the United States do you think survive at least 5 years?"	Percentages ranges (0-9%, 10-29%, 30-49%, 50-69%, 70-89%, and 90-100%)	Answers were considered "accurate," "overestimates," or "underestimates" in comparison to 78-80%	No*
*Question previously published				

© 2024 Chiu AS et al. *JAMA Network Open.*
