# Supplementary material for: Medical Maximizing Preferences and Beliefs About Cancer Among US Adults
Source: JAMA Netw Open. 2024 Jun 14;7(6):e2417098. doi: 10.1001/jamanetworkopen.2024.17098 (PMC11179133; doi:10.1001/jamanetworkopen.2024.17098)
Supplement: Supplement 2. — Data Sharing Statement [file jamanetwopen-e2417098-s002.pdf]

Data Sharing Statement

Chiu. Medical Maximizing Preferences and Beliefs About Cancer Among US Adults. *JAMA Netw Open*. Published June 14, 2024. doi:10.1001/jamanetworkopen.2024.17098

Data
Data available: Yes
Data types: Deidentified participant data, Data dictionary
How to access data: scpitt@med.umich.edu
When available: With publication

Supporting Documents
Document types: None

Additional Information
Who can access the data: Researchers whose proposed use of the data has been approved
Types of analyses: For any purpose
Mechanisms of data availability: With signed data use agreement
Any additional restrictions: For 3 years following manuscript publication
